# The Frailty Risk Score predicts length of stay and need for rehospitalization after kidney transplantation in a retrospective cohort: a pilot study

**DOI:** 10.1186/s40814-019-0534-2

**Published:** 2019-12-10

**Authors:** Joanna Schaenman, Loren Castellon, Emily C. Liang, Deepa Nanayakkara, Basmah Abdalla, Catherine Sarkisian, Deena Goldwater

**Affiliations:** 1 Division of Infectious Diseases, Department of Medicine, David Geffen School of Medicine, Los Angeles, CA USA; 2 Albert Einstein College of Medicine, New York City, New York USA; 3 Division of Nephrology, Department of Medicine, David Geffen School of Medicine, Los Angeles, CA USA; 4 Division of Geriatrics, Department of Medicine, David Geffen School of Medicine, Los Angeles, CA USA

**Keywords:** Frailty, Kidney transplantation, Older, Comorbidity, Readmission

## Abstract

**Background:**

Frailty is a widely used measure in older patients as a predictor of poor outcomes after hospitalization and surgery. There is a growing body of data in kidney transplantation suggesting frailty can predict adverse outcomes. There is interest in using chart review measures of frailty and multimorbidity, as they may be equally predictive as physical measurement. This approach holds promise for patient evaluation, identifying candidates for prehabilitation, and targeting resources towards those anticipated to have an increased rate of clinical challenges after kidney transplantation. Frail patients who are often older may place a large resource and economic burden on transplant programs.

**Methods:**

We applied a previously published chart review–based approach in a retrospective, pilot study to calculate the Frailty Risk Score (FRS) utilizing a cohort of kidney transplant patients. We reviewed concurrent comorbidities using the Charlson comorbidity (CM) score to determine the feasibility and utility of applying this approach in transplant patients to predict post-transplant outcomes such as length of hospitalization and the need for rehospitalization.

**Results:**

Sixty kidney transplant recipients were evaluated by chart review, 23 characterized as older (> = 60) and 37 younger (ages 30–59). Median FRS score was 3 (range 1–7). Higher FRS was significantly associated with increased patient age (high FRS 19% in younger patients, 43% in older patients). Increased CM score was also associated with increased patient age. Patients with a high FRS stayed in the hospital for an average of 8 days, compared with 5.7 days for a low FRS. Patients with high FRS were readmitted an average of 2.9 times compared with an average of 1.1 for those with a low FRS. FRS score remained significant for predicting outcomes after adjustment for patient age.

**Conclusion:**

Elevated FRS prior to transplantation was associated with increased hospital stay and the need for readmission in kidney transplant recipients. This analysis demonstrates the potential strength of chart review in evaluating frailty prior to transplantation, permitting risk stratification and targeting of resources for rehabilitation and close post-transplant monitoring. Frail patients may benefit from targeted “prehabilitation” to attenuate the associated adverse clinical outcomes.

## Background

The number of older adults with end-stage renal disease (ESRD) continues to grow, with the highest incidence and prevalence of ESRD in patients over age 65 leading to an increased number of older transplant recipients over age 50 [1]. Older patients have higher pre-transplant mortality rates compared with younger patients, higher mortality rates and rates of loss of allograft function after transplantation [1]. However, older patients have superior outcomes after kidney transplantation compared with remaining on dialysis [2], and modern outcomes for older patients are superior compared with previous eras, although the discrepancy in outcomes compared with younger transplant recipients continues [3]. Analysis of data from the United Network for Organ Sharing suggests that post-transplant outcomes vary substantially within the older patient population [2]. This observation suggests that additional evaluation of older patients would have important utility in identifying older patients at increased risk for adverse outcomes after transplantation. However, although tools for predicting kidney allograft outcomes such as the Kidney Donor Profile Index and Estimated Post Transplant Survival (EPTS) score have been developed, there currently does not exist a comprehensive clinical tool for predicting patient outcomes after transplantation, especially for older patients. The EPTS score incorporated into the current United Network for Organ Sharing (UNOS) Kidney Allocation System includes only age, time on dialysis, presence or absence of diabetes, and history of previous transplant, with the goal of allocating higher quality kidney to recipients who have a longer estimated survival post-transplant. Improvements in the prognostication of this tool would improve organ allocation and prevent unfair discrimination against chronologically older transplant candidates who would otherwise be predicted to do well after transplantation [2].

Approaches previously utilized in evaluating older patients to predict adverse clinical outcomes prior to other surgical procedures include frailty and assessments of multimorbidity [4, 5]. Frailty is defined as a decrease in physiologic reserve leading to an increased vulnerability to adverse events after a medical stressor [5]. Multiple frailty assessment tools have been developed, as evaluation by the so-called “eyeball” test is subjective and therefore not an appropriate approach for candidate selection [2]. One common tool, the Fried frailty phenotype, measures walk speed, grip strength, and patient-reported attributes of weight loss, caloric expenditure, and exhaustion [4]. Another tool, the short performance physical battery (SPPB), focuses on physical performance aspects frailty including walk speed, balance, and lower extremity strength [6]. There are extensive publications supporting the value of these measurements in predicting hospitalization and death in older patients, although this approach is relatively new in the transplant world [7–9]. In addition to metrics based on direct measurement of frailty, there has also been the development of a “frailty index” and measurements of comorbidity, sometimes described as the accumulation of multiple deficits, which have been shown to correlate with frailty phenotypes and have similar predictive value [10]. The Charlson comorbidity (CM) index is a well-described approach for measuring multimorbidity, which has been well-validated in multiple cohorts of older patients [11]. Lekan et al. [12] published a Frailty Risk Score (FRS) which demonstrated excellent predictive ability for rehospitalization and death in older patients. Based on components of frailty phenotypes described in prior research, consensus opinion, and practice guidelines, the FRS is composed of 16 biopsychosocial risk factors that are available in the electronic medical record including fatigue, weakness, dyspnea, chronic pain, falls, vision impairment, urinary incontinence, and nutrition issues plus four commonly measured biomarkers, namely, C-reactive protein, white blood cell count, hemoglobin, and albumin. Given that this assessment is based on electronic healthcare record review, it is therefore very well suited for retrospective review of patient outcomes. In addition, the limited number of variables required for FRS assessment makes it more attractive for clinical implementation compared with other chart-based frailty indices, which may require a review of over 70 clinical variables [5].

Other tools for assessing physical frailty and comorbidity have now been applied to kidney transplant candidates and recipients for the prediction of adverse outcomes after kidney transplantation, with a small but growing body of evidence supporting its utility. Chronic kidney disease is associated with frailty in older patients, and previous studies have found an association with declining kidney function and frailty by SPPB [13]. The prevalence of frailty by Fried frailty testing was 19.5% in one large cohort, with increased association with increased recipient age and predicting the increased length of hospital stay, need for rehospitalization, and increased risk of death after kidney transplantation [14–16]. The CM index has also been shown to be associated with mortality after kidney transplantation in one cohort study from Italy [17]. In addition to measuring physical frailty and comorbidities, other geriatric assessments of transplant patients have shown promise for predicting outcomes in patients with chronic kidney disease, including measurement of activities of daily living (ADLs) and cognitive function [18, 19], supporting a more comprehensive assessment including frailty as well as other markers of geriatric disability in older transplant candidates and recipients. Although tools exist in kidney transplant for predicting allograft function, prediction of other clinical outcomes remains imprecise. Given the advancing age of transplant candidates, additional research exploring alternate methods for patient evaluation and risk stratification prior to transplantation is necessary.

Frailty and other measurements of geriatric impairment are of special interest in the context of transplantation given their association with “immunologic frailty,” represented by linked but opposing characteristics of immune senescence and pro-inflammation [20, 21]. In fact, a small number of studies have shown a link between physical frailty and inflammation in transplant candidates or recipients in the setting of both lung and kidney transplantation [22, 23]. This suggests that the so-called “inflammaging” seen in the non-transplant population may persist in older patients after transplantation despite the receipt of immunosuppression [24–26]. Immune senescence has also been demonstrated both in older adults on dialysis and in kidney transplant recipients [27, 28], and may explain increased vulnerability to infection and malignancy observed in older transplant recipients [29].

Although objective assessment of frailty via in-person evaluations such as Fried and SPPB remains the cornerstones of geriatric assessment, electronic medical record–based evaluation offers many advantages in terms of reduced cost, manpower needs, and the ability to perform assessments retrospectively. Whether these evaluations may provide similar prognostic data for candidate assessment and post-transplant prediction of outcomes compared with objective frailty assessments remains incompletely characterized. We therefore sought to apply FRS and CM analysis approach using a medical record–based evaluation in a pilot study to determine whether pre-transplant assessments had the ability to predict post-transplant outcomes in this pilot study utilizing a previously characterized cohort of matched older and younger kidney transplant recipients [11, 12]. The primary objective of this study was to determine whether FRS and CM could be abstracted from the electronic medical record in kidney transplant candidates and to describe the mean and distribution of measured scores in patients with a range of chronologic ages. Secondary objectives were to determine whether FRS and CM were associated with adverse clinical outcomes including length of stay, need for rehospitalization, infection, and rejection. This work continues the small but growing trend of applying concepts from geriatric medicine to the evaluation of older transplant candidates, representing a new paradigm for patient evaluation and risk stratification in the field of transplantation.

## Methods

**Fig. 1:**
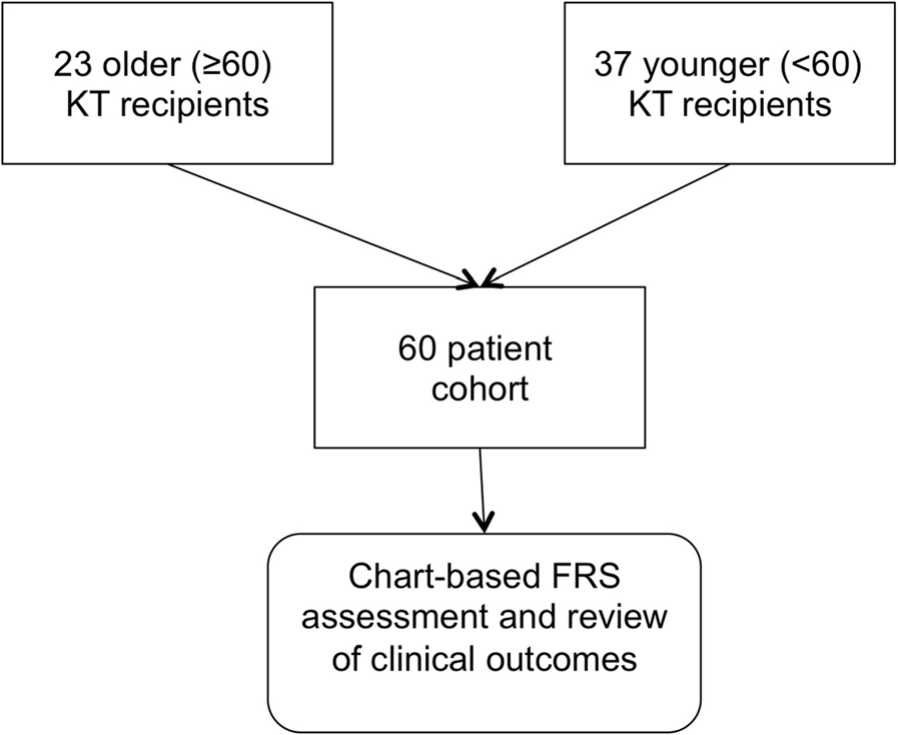
Flow diagram demonstrating the creation of study cohort. Older kidney transplant (KT) with samples available for immunologic assessment (*n* = 23) were cohort matched with a population of younger kidney transplant recipients (*n* = 37) by living versus deceased donor and induction immunosuppression (Table 1) as described previously [28]. Frailty Risk Score (FRS) and clinical outcomes were assessed based on a review of the electronic medical records as described in the Methods section

We retrospectively evaluated the electronic medical record in a previously well-characterized cohort of older and younger kidney transplant recipients propensity matched on induction type (anti-thymocyte globulin (ATG) versus basiliximab) and living versus deceased donor under the approval of the UCLA Institutional Review Board [26, 28] (Fig. 1). In brief, these were 22 older patients, defined as older than 60 years, who agreed to participate in the study, matched with 38 patients between the ages of 30 and 51 years, for whom blood samples collected 3 months after transplant were available, who underwent T cell, monocyte, and cytokine analysis [26, 28]. Age 60 was used to define older for the purpose of this analysis based on the precedent set by other analyses of older kidney transplant recipients [30–32]. Subjects were enrolled over a 2-year period (2014–2016). Patients were on similar protocolized regimens of tacrolimus, mycophenolate mofetil, and prednisone for the prevention of rejection, as described previously [26]. Given that this analysis was based on a predetermined set of patients, a power calculation was not performed.

To determine whether a chart-based review of records was feasible for predicting post-transplant outcomes, the CM score and FRS were calculated based on evaluations by transplant nephrology, social work, psychiatry, and other healthcare professionals at the time of initial evaluation for transplant listing as previously described [11, 12]. The first year after transplantation was reviewed for the incidence of biopsy-defined rejection and infection including invasive infection and CMV viremia as described [28]. In addition, the electronic medical record was reviewed to determine the length of the initial hospitalization for transplantation, number of readmissions during the first year, and incidence of patient death. The FRS was defined as “high” (> 3) or “low” (≤ 3) as the median score for the cohort was 3. Similarly, the CM score was defined as “high” (> 2) or “low” (≤ 2).

Statistical analysis was performed using JMP Pro 12 (SAS Software). Pearson chi-square test was used to compare categorical variables. Differences between continuous values were compared by *t* test. Multivariate logistical regression was performed by stepwise regression using minimum BIC as a stopping rule adjusted for patient age to avoid the confounding effect of age on patient outcomes. ROC curve was calculated by logistic regression using the whole model test to determine the predictive value of FRS for hospital readmission. Given that this was a pilot study with a primary objective of feasibility, a target AUC score was not pre-specified.

## Results

### Demographics of the patient cohort

Twenty-three older (≥ 60 years old) and 37 younger (ages 30–59 years old) kidney transplant patients from our center were evaluated (see Table 1 and Fig. 1). These patients had similar frequencies of sex, race, and ethnicity, and were propensity matched on factors known to influence outcomes after kidney transplantation, namely, use of ATG for induction immunosuppression and donor type, as described previously [26].

**Table 1:** Demographic and clinical characteristics and of older compared with younger kidney transplant recipients

	Younger (< 60) *n* = 37	Older (≥ 60) *n* = 23
Median age (range)	43 (34–51)	67 (60–80)
Male sex	60%	74%
White race	68%	65%
Hispanic	41%	35%
Dialysis pre-transplant	73%	91%
Diabetes pre-transplant	32%	57%
Induction, ATG	30%	30%
Deceased donor	46%	44%
Tacrolimus Y/N*	95%	83%
Tacrolimus trough (mean, SD)*	9.7 (3.3)	10.1 (3.6)
MMF daily dose in g (mean, SD)	1.4 (0.7)	1.2 (1.2)
Prednisone daily dose in mg (mean, SD)	5.3 (1.6)	5.6 (3.1)

*Immunosuppression drugs and troughs assessed at 3 months post transplant

### Results of FRS analysis

For the primary study objective, we found that FRS was able to be easily calculated from a review of clinic notes from the date of evaluation for transplant suitability, including a review of nephrologist, dietician, and social worker notes. This analysis took approximately 15 min for each patient.

**Fig. 2:**
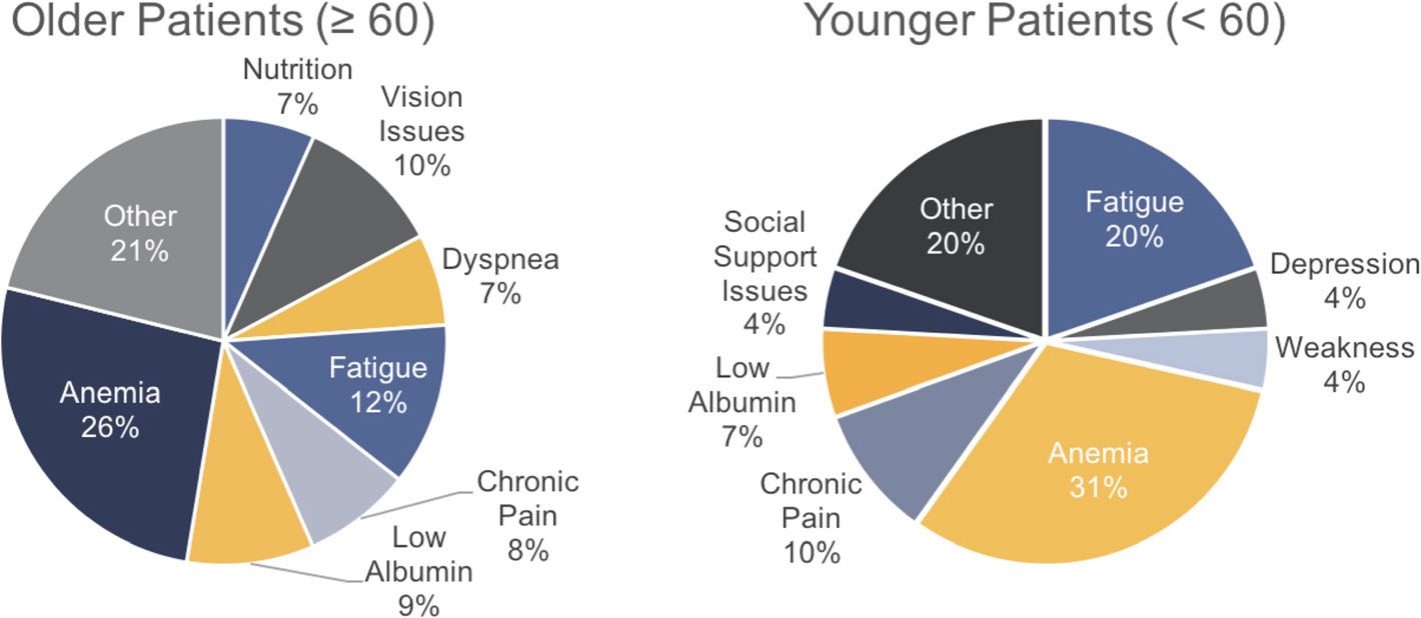
Distribution of Frailty Risk Score (FRS) variables in older compared with younger patients. Pie chart demonstrates relative percentages of FRS components for older (*n* = 23) and younger (*n* = 37) patients

Older patients were more likely to have increased in FRS due to problems with nutrition, vision issues, and dyspnea, compared with younger patients (Fig. 2), although these differences did not reach statistical significance. Younger patients were more likely to have fatigue as a contributor to FRS. In contrast, both older and younger patients had a similar incidence of pain, depression, and weakness.

Patients were categorized as high or low FRS (> 3 or ≤ 3) as described above: In the total cohort, the mean FRS was 3.02 (standard deviation (SD) 1.4) and median score was 3, ranging from 1 to 7. Twenty-eight percent of the total cohort was characterized as having a high FRS (17/60). Many older and younger patients had FRSs influenced by anemia, chronic pain, or the report of fatigue on evaluation (Fig. 2).

### Frailty or multimorbidity score and patient demographic characteristics

**Fig. 3:**
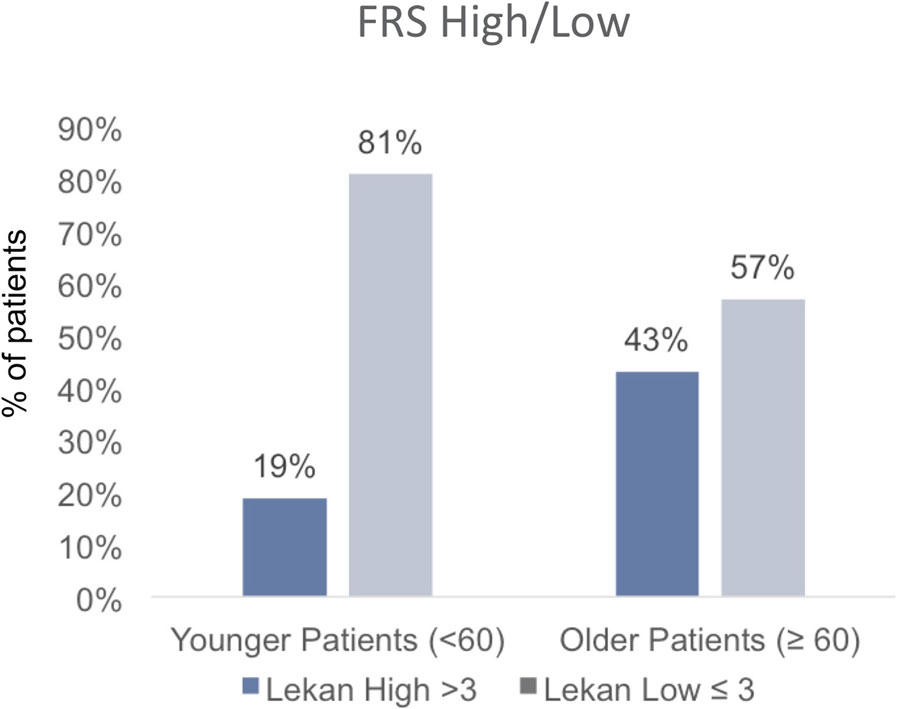
Proportion of high and low Frailty Risk Score (FRS) between younger and older patients. Bar graphs show the distribution of FRS High (dark bar) and FRS Low (light bar) within younger and older patients. Statistical analysis by Pearson’s chi-square test

**Fig. 4:**
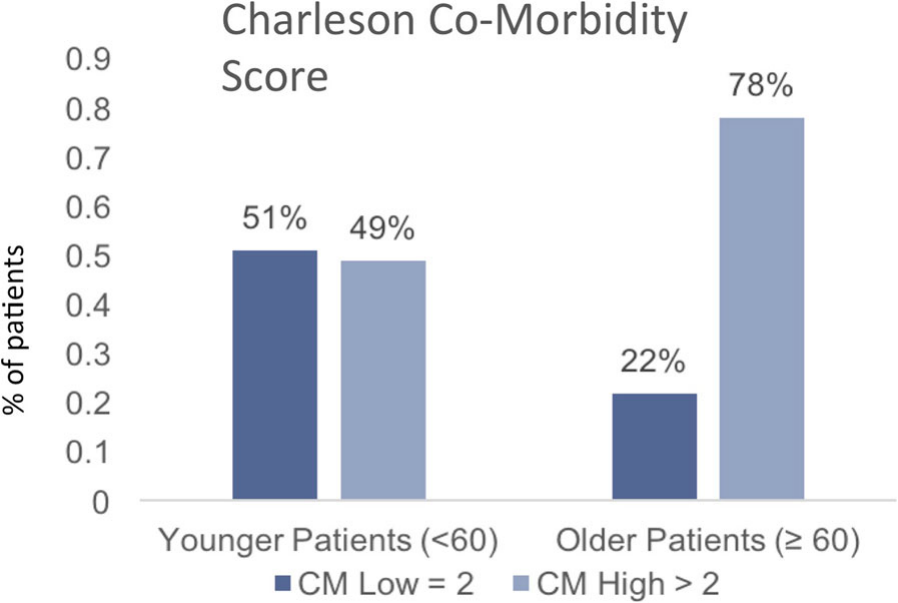
Proportion of high and low Charleson comorbidity (CM) index between younger and older patients. Bar graphs show the distribution of CM High (dark bar) and CM Low (light bar) within younger and older patients. Statistical analysis by Pearson’s chi-square test

Evaluation of high versus low FRS was associated with patient age (characterized as young v. old), with a high FRS in 19% of younger patients, compared with 43% in older patients (Fig. 3). The CM also associated with patient age, with 49% of younger patients having high (> 2) CM while 78% of older patients had high CM (Fig. 4).

There was no significant association observed between sex and FRS—38% of women had a high FRS, compared with 23% of men. There was also no significant difference in FRS by race or ethnicity (data not shown). There was similarly no association between sex, race, or ethnicity and CM.

No significant association was observed between those ever on dialysis before transplant by FRS. There was a trend towards increased FRS with diabetes, with 40% of those with diabetes having a high FRS compared with 21% of those without diabetes. However, there was no association between patient age and diabetes in our cohort, although there was a trend towards an increased incidence of pre-transplant diabetes in older patients. Not surprisingly, CM was associated with both dialysis and diabetes.

### Frailty or multimorbidity score and length of initial hospitalization

**Fig. 5:**
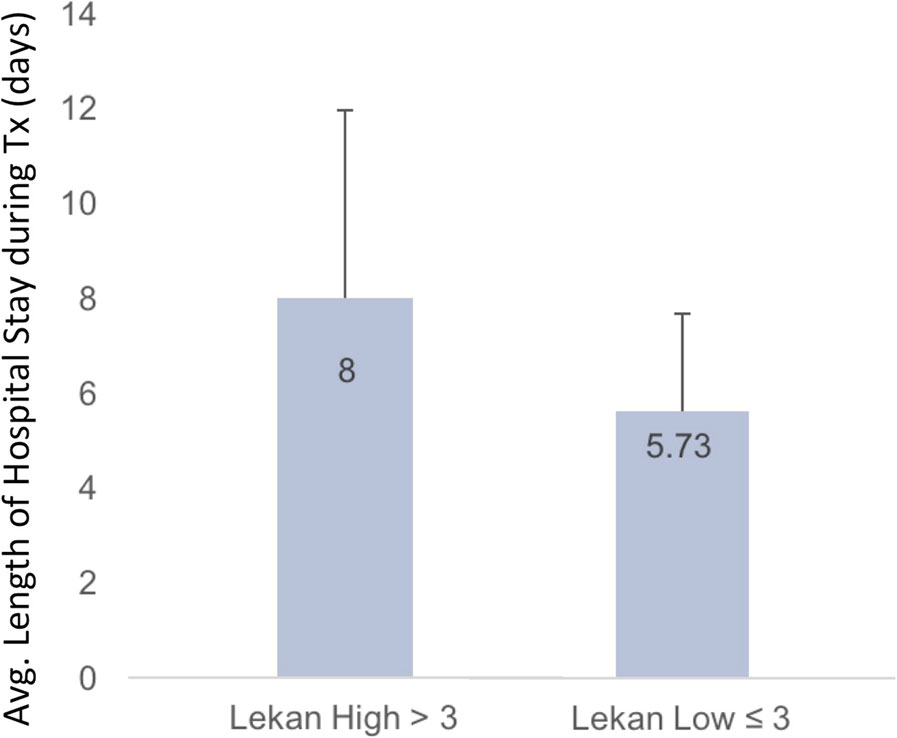
Association between high and low Frailty Risk Score (FRS) and length of hospital stay during transplantation (Tx). Bar graphs show the length of average (avg.) hospital stay in days by FRS High (dark bar) compared with FRS Low (light bar); standard deviation shown in error bar. Statistical analysis by *t* test

To address the secondary objective of assessing whether FRS was associated with adverse clinical outcomes, we evaluated for the association between length of stay and need for hospital readmission. Increased FRS at evaluation was found to predict a significantly longer post-transplant hospital course. Patients with a high FRS stayed in the hospital for an average of 8.0 (± 4.0) days, compared with 5.7 (± 2.1), days for a low FRS (CI − 4.39 to − 0.17) (Fig. 5). Linear analysis confirmed this finding, with a significant association observed between FRS and length of stay after transplant. Interestingly, analysis of FRS subcomponents demonstrated an association for one element, abnormal WBC. In contrast, older compared with younger patient age was not significantly associated with length of hospital stay, 5.8 ± 1.9 days for older compared with 7.5 ± 4.0 days for younger patients.

### Frailty or multimorbidity score and need for readmission

**Fig. 6:**
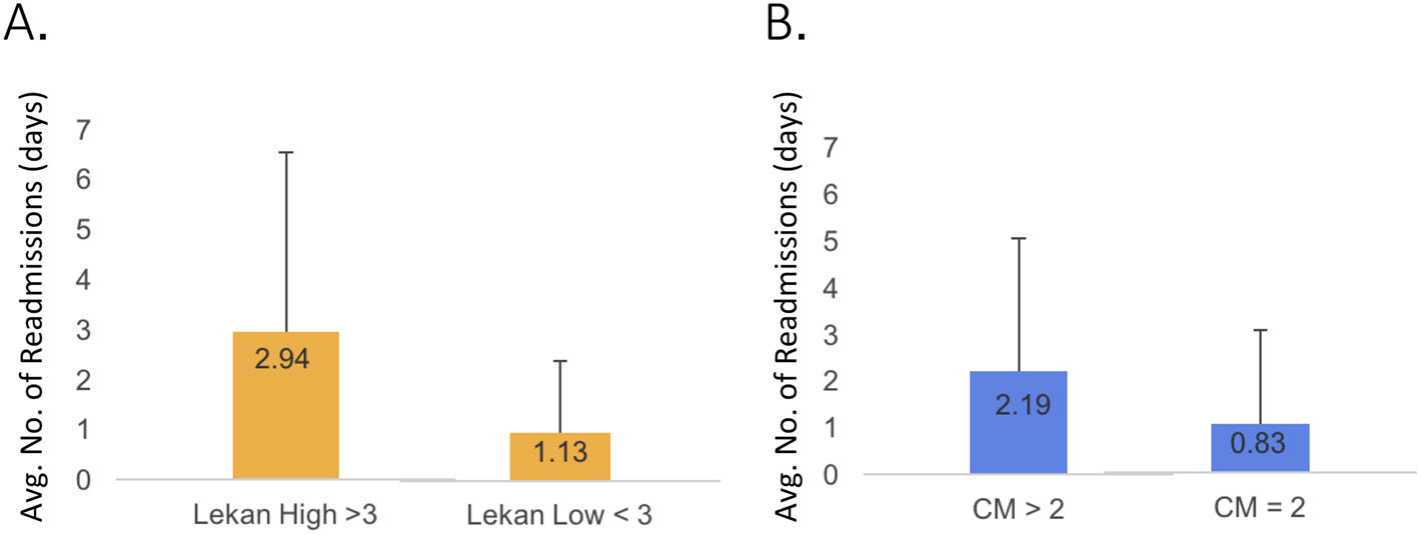
**a** Association between high and low Frailty Risk Score (FRS) and average (avg.) number of readmissions. Bar graphs show the average (avg.) number of hospital readmissions by FRS High compared with FRS Low; standard deviation shown in error bar. **b** Association between high and low Charleson comorbidity (CM) index and average (avg.) number of readmissions. Bar graphs show the average (avg.) number of hospital readmissions by CM High compared with CM Low; standard deviation shown in error bar. Statistical analysis by *t* test

Both the FRS and CM significantly predicted the average number of readmissions following transplantation (Fig. 6). The patients with a high FRS were readmitted an average of 2.94 times compared with an average of 1.13 for those with a low FRS (high FRS SD = 3.58, low FRS SD = 1.77; CI − 3.70 to 0.09). The patients with a high CM score were readmitted an average of 2.19 times, compared with an average of 0.83 for those with a low CM score (CM high SD = 2.87, CM low SD = 1.63; CI − 2.53 to −.19). There were not any FRS subcomponents that were predictive of the need for readmission when analyzed independently (data not shown). Older compared with younger patient age was not significantly associated with the need for readmission.

ROC curve analysis of FRS and the need for readmission resulted in an AUC of 0.57. This was similar to the analysis of CM and the need for readmission, which resulted in an AUC of 0.62, while the AUC for patient age and readmission was 0.58.

In an exploratory multivariate analysis to predict the number of readmissions including both FRS and patient age old/young, only FRS remained statistically significant, with *p* = 0.004 for FRS high/low and *p* = 0.080 for patient age old/young, suggesting that the association with frailty is independent of patient age. We also observed in a multivariate model that FRS maintained statistical significance in combination with patient age to predict the length of post-transplant hospitalization, with *p* = 0.017 for FRS and 0.097 for patient age. Similarly, CM and patient age in model together demonstrated CM statistical significance (*p* = 0.016) and patient age not significant (*p* = 0.105). Neither CM nor patient age was significantly associated with time to post-transplant readmission.

### Frailty or multimorbidity score and infection or rejection

As an additional secondary objective, we evaluated whether FRS was associated with infection or rejection. We observed a trend towards increased FRS and development of invasive infection in the first year after transplant, with 70% of patients developing infection having a high FRS, compared with 46% of patients who did not develop invasive infection in the first year post transplant. No significant association was seen between the FRS and CMV viremia in the first year post transplant (data not shown). Similarly, in this cohort, there was no association between FRS and acute rejection in the first year after transplantation. As only one patient in the cohort died during the first year post transplant, so we were not able to investigate an association between mortality and frailty assessment.

## Discussion

In this pilot study of kidney transplant recipients assessed at the time of transplant evaluation, we found that it was possible to assess FRS and CM based on the review of the electronic medical record. In addition, there was a suggestion that these indices had the ability to predict adverse clinical events post transplant. This fills an unmet need in the field given the current absence of tools for predicting clinical outcomes including length of stay after transplantation and allows for planning of a future study to validate the findings of this pilot investigation. Although the observation that the FRS score was associated with patient age is not a surprising observation, it is notable that a significant number of younger patients had high FRS, and a significant number of older patients have low FRS, suggesting that chronologic age alone is not sufficient for patient evaluation. Evaluation of the FRS score was straightforward to perform and was not time consuming, suggesting feasibility for routinely applying this analysis to transplant candidate evaluation. As computer-assisted techniques become more available, it may become possible to perform assessments in an automated fashion, saving additional time.

Our data suggests that the assessment of these measures at the time of candidacy evaluation and listing may help identify at-risk patients. The goal of this type of pre-transplant assessment is not to exclude patients from consideration for transplantation, but rather to direct clinical resources towards patients at increased risk for adverse outcomes in order to improve outcomes. Potential approaches to achieve this goal would include prehabilitation, where targeted training is directed at frail patients to attempt to improve physical strength and resilience prior to the stressor of transplantation, which has shown promise in pilot studies [33]. In addition, given the known correlation between immune dysfunction and frailty, patients identified as frail could receive individualized immunosuppression to try to prevent post-transplant adverse events, with decision-making performed in concert with the assessment of the immunologic risk of donor and recipient. Finally, additional attention could be directed towards more vulnerable patients post-operatively and peri-discharge to improve transitions of care and minimize readmission.

The mechanism of frail patients experiencing longer hospital stays and the need for readmission may be related to increased vulnerability to stressors and the inability to regain equilibrium after surgery and immunosuppression start. This fits with our understanding of frailty as a multisystem dysfunction of decreased physical resilience and suggests that our chart-based FRS is able to assess some of the same biologic measures as objective testing such as FFP or SPPB. Future studies should assess translational markers of immune dysfunction such as cellular senescence and inflammation to determine if there is an association with FRS to try to determine the underlying mechanisms underlying frailty.

The challenge of frailty in transplant patients is likely to increase given the increasing prevalence of ESRD in older patients [34]. This trend has led to an increase in the age of patients undergoing kidney transplantation [1]. In addition to increased age, the incidence of physical frailty in patients awaiting transplantation is high as measured by FFP, and many of these patients also have comorbid conditions that may have led to exclusion from consideration for transplantation in previous eras. This makes the development of methods for pre-transplant candidate assessment and prediction of adverse clinical outcomes increasing important. The potential importance of frailty in the evaluation of transplant candidates has recently been emphasized in a statement from the American Society of Transplantation [35].

Limitations of this study include the relatively small cohort size and the cross-sectional design. In addition, a priori definition of feasibility was not established prior to the start of the study, limiting our ability to objectively assess the attainment of this endpoint. However, as a pilot study, we have gathered data supporting the potential value of this approach that can lead to additional study in a larger patient cohort. One benefit of the single-site design is the similarity in terms of evaluation, surgical procedure, and post-transplant medication titration seen with a single transplant center protocol. A limitation of analysis of this patient population in terms of CM index assessment is that all patients by definition have ESRD, one of the CM components, decreasing the discriminatory ability of this index.

Future studies will attempt to validate these findings in a larger patient cohort to determine the reliability of the FRS for pre-transplant patient evaluation. In addition, we will evaluate FRS after transplantation to determine if there is evidence of the impact of kidney transplantation in the early (3 months) and late (12 months) periods after transplant. We additionally propose to assess additional chart-based frailty tests as well as for concordance with physical assessments FFP and SPPB, and sarcopenia as measured by psoas muscle mass. In addition, as described above, we will evaluate the association between FRS and markers of T cell dysfunction such as senescence and exhaustion as well as pro-inflammatory innate immune cells and cytokines to try to evaluate mechanisms behind the development of frailty. New approaches utilizing artificial intelligence (AI)–based approaches for the prediction of clinical outcomes may be especially well suited for application to the measurement of a chart-based frailty index such as the FRS.

Measurement of frailty by chart-based assessment such as FRS may well become standard care for pre-transplant evaluation and post-transplant monitoring as these tools are more strongly associated with adverse post-transplant outcomes than chronologic age. The addition of frailty testing to a comprehensive clinical assessment of transplant candidates holds promise for improving candidate selection, and for risk stratification to improve post-transplant outcomes. Given the association between frailty and post-transplant survival, such a chart-based tool may have utility in organ allocation policy, as its addition to the current EPTS score would likely provide a more comprehensive assessment of recipient survival than EPTS score alone, which accounts for only four factors including recipient age (https://optn.transplant.hrsa.gov/media/1511/guide_to_calculating_interpreting_epts.pdf). A chart-based review tool has the advantage of potentially being able to be calculated by a transplant coordinator and entered in the organ allocation computer system to support medical decision-making. Additional studies will shed more light on the mechanism behind adverse outcomes and frailty in transplant candidates and recipients.

## Conclusion

Chart-based frailty review provides a potential opportunity to risk stratify kidney transplant candidates prior to transplantation to anticipate post-transplant complications such as length of hospital stay and need for readmission. This information can potentially target interventions to high-risk patients, whose risk for adverse clinical outcomes would not be able to be predicted by age or other clinical characteristics alone.

## Data Availability

The datasets analyzed for this study are not publicly available given the existence of multiple identifying patient characteristics; however, datasets are available from the corresponding author on reasonable request.
